# A Comprehensive Scoping Review on Diet and Nutrition in Relation to Long COVID-19 Symptoms and Recovery

**DOI:** 10.3390/nu17111802

**Published:** 2025-05-26

**Authors:** Galya Bigman, Marius Emil Rusu, Nicole Shelawala, John D. Sorkin, Brock A. Beamer, Alice S. Ryan

**Affiliations:** 1 Division of Gerontology, Department of Epidemiology and Public Health, University of Maryland School of Medicine, Baltimore, MD 21201, USA; jsorkin@som.umaryland.edu; 2 Department of Pharmaceutical Technology and Biopharmaceutics, Faculty of Pharmacy, Iuliu Hatieganu University of Medicine and Pharmacy, 400012 Cluj-Napoca, Romania; rusu.marius@umfcluj.ro; 3 Health Sciences and Human Services Library, University of Maryland, Baltimore, MD 21201, USA; nshelawala@hshsl.umaryland.edu; 4 Baltimore Veterans Affairs Medical Center, Division of Gerontology, Geriatrics and Palliative Medicine, Department of Medicine, University of Maryland School of Medicine, Baltimore, MD 21201, USA; bbeamer@som.umaryland.edu (B.A.B.); aryan@som.umaryland.edu (A.S.R.); 5 Baltimore Geriatric Research, Education and Clinical Center, Veterans Affairs Maryland Health Care System, Baltimore, MD 21201, USA

**Keywords:** post-acute COVID-19 syndrome, nutrients, fatigue, gut microbiota, nutraceuticals, bioactive compounds, biomarkers, vitamins, supplements, SARS-CoV-2

## Abstract

**Background/Objectives:** Long COVID-19 is characterized by persistent symptoms lasting three months or more following SARS-CoV-2 infection. Nutrition has emerged as a modifiable factor influencing recovery trajectories and symptom burden; however, existing evidence remains fragmented across diverse study designs and populations. This scoping review synthesized global evidence on the role of diet and nutrition in managing long COVID-19 symptoms and supporting recovery. **Methods:** Following PRISMA-ScR and Joanna Briggs Institute guidelines for scoping reviews, we searched major biomedical databases for studies published between 2020 and 2025. Eligible studies examined dietary intake, nutritional status, or nutrition-related interventions in adults with long COVID-19. **Results:** After duplicates were removed, 1808 records were screened, resulting in 50 studies that met the inclusion criteria—27 intervention studies and 23 observational studies. Nutritional exposures included micronutrients (e.g., vitamins D, K_2_), amino acids (e.g., L-arginine), multinutrient formulations, microbiota-targeted therapies (e.g., probiotics, synbiotics), nutritional status, diet quality, and whole-diet patterns (e.g., the Mediterranean diet). Approximately 76% of studies reported improvements in long COVID-19-related symptoms such as fatigue, mood disturbances, physical function, and markers of inflammation. **Conclusions:** Diet and nutrition may support long COVID-19 recovery by targeting inflammation and the gut microbiome to alleviate symptoms and improve functional outcomes. Well-powered trials of whole-diet approaches, combined with targeted supplementation, are needed to confirm their potential as scalable, accessible tools for post-COVID-19 recovery and management.

## 1. Introduction

As the acute phase of the COVID-19 pandemic waned—due to widespread vaccination, population-level immunity, and the emergence of less virulent SARS-CoV-2 variants—attention shifted toward a new and enduring public health challenge: long COVID-19, also known as post-acute COVID-19 condition (PCC) [1,2,3,4]. The cumulative global incidence of long COVID-19 is estimated at around 400 million individuals [5]. This condition is characterized by a broad spectrum of persistent manifestations—including fatigue, muscle weakness, cognitive impairment, gastrointestinal disorders, and respiratory difficulties—that may persist for months or years, irrespective of the initial illness’s severity [5].

Among affected individuals, diet-related health is often compromised due to both physiological dysfunction and enduring clinical complaints. Damage to organs such as the gastrointestinal tract, liver, and vasculature may impair nutrient absorption, alter metabolic processes, and increase physiological demands [5,6,7,8]. These disruptions are further exacerbated by chronic inflammation, immune dysregulation, and imbalances in the gut microbiota, all of which play central roles in maintaining homeostasis [6,7,8]. In addition, long COVID-19 has been associated with virus-induced metabolic reprogramming, characterized by alterations in energy pathways, mitochondrial dysfunction, oxidative stress, and impaired host defenses [9]. Symptoms such as altered taste and smell, digestive discomfort, chronic cough, and excessive thirst may further suppress appetite and reduce intake [10,11], collectively delaying recovery and worsening nutritional deficits [11,12].

Initial research on COVID-19 and diet primarily focused on immune-supportive micronutrients—such as vitamins A, B-complex, C, D, K, zinc, and selenium—highlighting their role in moderating acute disease severity [13,14,15]. However, growing evidence suggests that addressing the long-term consequences of infection requires a broader nutritional perspective. Increasing attention has shifted toward multi-nutrient combinations, functional foods, bioactive compounds, and microbiota-modulating strategies, alongside dietary patterns such as plant-based and Mediterranean diets [12,16,17,18,19,20,21]. Despite these promising developments, the evidence base remains fragmented, with few high-quality studies to guide practice and many unanswered questions regarding optimal dietary approaches [16,17,18,19,22,23].

Given the prolonged and multifaceted nature of long COVID-19, and the absence of curative treatments, care remains largely supportive, highlighting the need to synthesize existing evidence on diet- and nutrition-related strategies to guide future research and clinical practice [24]. To fill this gap, this scoping review examines the existing literature on dietary and nutritional factors in relation to long COVID-19 symptoms and recovery. Drawing from population-based studies—including both observational and interventional designs—it synthesizes evidence on individual nutrients, whole-diet patterns, microbiome-targeted strategies, and nutrition components embedded within multidisciplinary interventions. By mapping the scope and diversity of available evidence, this review identifies dietary and nutritional considerations relevant to symptom management and aims to inform future research priorities and evidence-based care strategies for individuals affected by long COVID-19.

## 2. Materials and Methods

### 2.1. Protocol and Registration

A review protocol was developed in collaboration with a research librarian (N.S.) at the University of Maryland, Baltimore. This scoping review follows the PRISMA-ScR guidelines [25] and the Joanna Briggs Institute Manual for Scoping Reviews [26]. A scoping review was chosen because the literature on diet and long COVID-19 is preliminary and expanding, characterized by methodological diversity, a broad conceptual scope, and the use of varying definitions for long COVID-19—conditions that preclude the focused questions and standardized quality appraisal required for systematic reviews or meta-analyses. In contrast, the scoping approach allows for comprehensive mapping of the existing evidence, identification of research gaps, and guidance for future studies.

### 2.2. Information Sources

A comprehensive search strategy was developed with the assistance of a professional librarian (N.S.). Information sources included the following electronic databases: Medline, PubMed, EBSCO, Cochrane Library, Embase, Scopus, the National Network of Libraries of Medicine (NNLM) resource, and the Cochrane Clinical Trials Registry. The initial search was conducted on 12 September 2024, and the most recent search was completed on 31 January 2025. In addition to database searches, manual searches were performed in selected review articles, and the reference lists of relevant studies were screened to identify additional sources. No authors were contacted for unpublished data.

### 2.3. Search Strategy

The search strategy combined Medical Subject Headings (MeSH) and free-text terms to ensure broad coverage. Core terms included the following: Long COVID-19-related terms: “long COVID”, “post-acute COVID-19 syndrome”, “post-COVID condition”, “post-COVID syndrome”, “chronic COVID”, and “COVID-19 survivors”. Diet-related terms: “diet”, “nutrition”, “dietary interventions”, “food intake”, “eating pattern*”, and “food*”. An example of the full search strategy used in Embase is the following: (((‘long covid’/exp OR (((‘coronavirus’ OR ‘ncov’ OR ‘cov’ OR ‘2019-ncov’ OR ‘sars-cov-2’ OR ‘sars-cov’ OR ‘covid19’ OR ‘covid-19′ OR ‘covid’) NEAR/2 (long OR chronic OR ‘post-acute sequela*’ OR sequela*)) OR ((‘post covid’ OR ‘post acute covid’ OR ‘post sars-cov-2’) NEAR/3 (syndrome* OR fatigue OR impairment* OR condition* OR infection* OR symptom* OR disorder* OR complication*))):ti,ab,kw) AND (‘nutrition’/exp OR ‘vitamin’/exp OR (vitamin* or diet* or nutrition* or nutrient* or micronutrient* or food or eating):ti,ab,kw)) NOT (‘juvenile’/exp NOT ‘adult’/exp)) NOT ((‘animal’/exp or ‘nonhuman’/exp) NOT ‘human’/exp).

This strategy was adapted for each database searched, as described above. In addition to database searches, the LitCovid portal [27] (https://www.ncbi.nlm.nih.gov/research/coronavirus/ Accessed 12 September 2024) was used as a supplementary source. LitCovid is a curated literature hub from the U.S. National Library of Medicine, National Institutes of Health, Bethesda, MD, USA that uses machine learning to classify COVID-19-related publications. At the time of search, it listed approximately 16,780 publications under the “Long COVID” filter. These results were screened using predefined diet-related terms. All records were imported into Covidence Systematic Review Software. Veritas Health Innovation, Melbourne, Australia, May 2025, www.covidence.org [28] for de-duplication, screening, and full-text review based on predefined eligibility criteria (Table 1).

### 2.4. Terminology and Definitions of Long COVID-19

Although “long COVID” is now the preferred term, earlier studies used alternative terminology reflecting the evolving understanding of the condition. This review includes studies that examined persistent, post-acute symptoms consistent with current definitions, typically requiring symptoms to last at least three months. Studies without a specified duration or with durations under three months were included if they clearly addressed ongoing health effects beyond the acute phase and focused on symptom profiles characteristic of long COVID-19.

### 2.5. Eligibility Criteria

All citations were stored in the Covidence reference manager [28]. Eligibility criteria were developed by G.B., N.S., and M.E.R. and are detailed in Table 1.

### 2.6. Selection of Sources of Evidence

**Figure 1:**
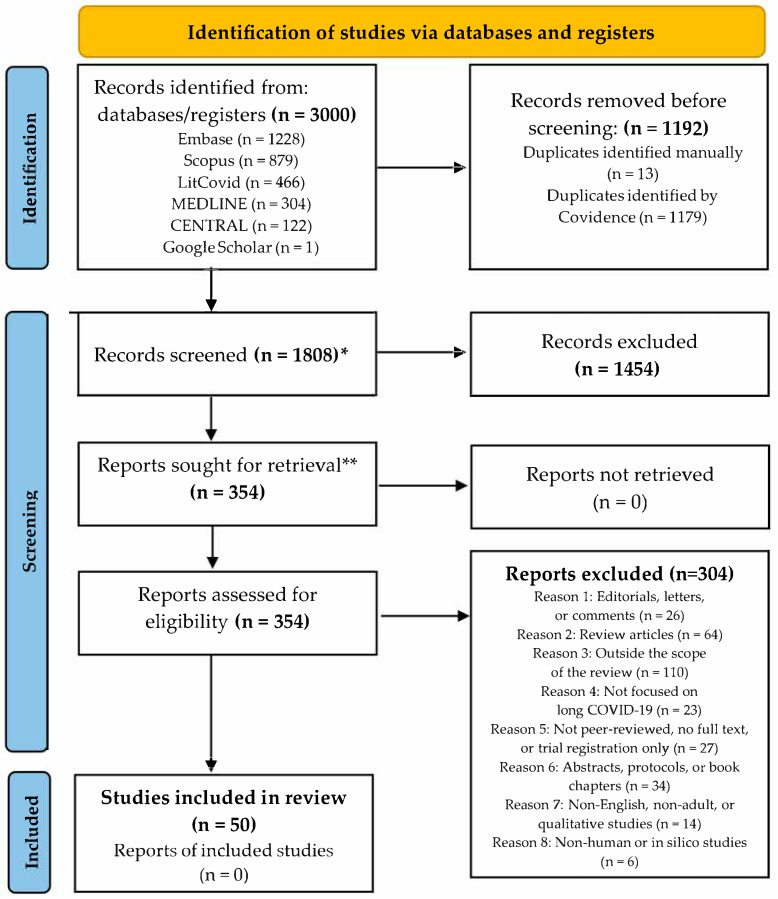
Study selection process illustrated via PRISMA 2020 flow diagram. * Title and abstract screening. ** Full-text review. Screening and data management were performed using Covidence (www.covidence.org).

Articles retrieved through the systematic search were imported into Covidence, and duplicates were removed. Title and abstract screening was conducted twice by a single reviewer (G.B.). The initial screening, conducted in September 2024, used a structured three-option system (“yes”, “no”, or “maybe”) designed to ensure inclusivity. In May 2025, a second round of screening was conducted on all previously excluded studies by the same reviewer (G.B), using the same eligibility criteria, to minimize the risk of omitting potentially relevant studies and to reduce selection bias [25,26]. Abstract and full-text screening were independently performed by two reviewers (G.B. and M.E.R.) using predefined criteria. Discrepancies were resolved through discussion. The study selection process was documented using the PRISMA flowchart in Figure 1.

### 2.7. Critical Appraisal of Sources of Evidence

In accordance with the Joanna Briggs Institute methodology for scoping reviews and the PRISMA-ScR guidelines [26], this review did not include a formal critical appraisal of individual sources of evidence. The principal objective of a scoping review is to map the breadth, scope, and key characteristics of existing research, rather than to evaluate study quality or assess risk of bias. However, to further assist readers in contextualizing the evidence, we applied a simplified ordinal rating to characterize the relative strength of each study. This descriptive classification—used solely for interpretive purposes—categorized studies as having low, moderate, or high strength based on study design, sample size, and overall methodological rigor, as outlined below:

Low strength: typically, small sample sizes (fewer than 100 participants), cross-sectional or open-label designs, and limited methodological or outcome reporting.

Moderate strength: generally sample sizes between 100 and 500, prospective cohort or well-reported observational designs, with defined outcomes, though some methodological limitations were present.

High strength: randomized controlled trials (RCTs) or large observational studies (often exceeding 500 or 1000 participants) with rigorous methodology, comprehensive reporting, and minimal risk of bias.

These ratings were not used to determine study inclusion but rather to enhance understanding of the nature and robustness of the existing evidence base.

### 2.8. Data Charting Process

An electronic data extraction form was developed to systematically collect information from each included study. Data extraction was conducted independently by two reviewers (G.B. and M.E.R.), with discrepancies resolved through discussion. No additional data were sought from study authors.

### 2.9. Data Items

Data were systematically extracted from each included study and organized according to key methodological and content domains. Extracted information included the following: citation details (Author, study year, reference number), country of origin, study design, and study sample characteristics (including sample size, mean age with standard deviation or median with interquartile range, if available, and sex distribution). We documented how long COVID-19 was defined or assessed, the specific type of nutritional exposure (e.g., biomarker, supplementation, diet pattern), and the long COVID-19-related outcomes examined. We also captured the tools or criteria used for outcome assessment, the main results, and an indication of statistical significance.

### 2.10. Synthesis of Results

Data were synthesized descriptively and thematically to identify patterns across study designs, nutritional exposures, and long COVID-19-related outcomes. Studies were grouped by type of nutritional exposure (e.g., specific nutrients, multinutrient or nutraceutical supplements, gut microbiota approaches). No statistical meta-analysis or critical appraisal was conducted, consistent with scoping review methodology [26]. To quantify the amount of significant evidence, we counted a study as having a significant finding if it reported at least one primary, statistically significant association between dietary or nutrition-related exposure and a long COVID-19 outcome.

### 2.11. Use of Large Language Models

ChatGPT-4.0 was used to refine the manuscript text drafted by the authors. The prompt provided was “Improve the text below for clarity, coherence, flow, and grammar for an academic audience” [29].

## 3. Results

### 3.1. Selection of Sources of Evidence

Of 3000 records identified, 1192 duplicates were removed, leaving 1808 for screening. After reviewing 354 full-text articles, 50 met inclusion criteria [19,30,31,32,33,34,35,36,37,38,39,40,41,42,43,44,45,46,47,48,49,50,51,52,53,54,55,56,57,58,59,60,61,62,63,64,65,66,67,68,69,70,71,72,73,74,75,76,77,78]. The selection process is shown in Figure 1.

### 3.2. Characteristics of Population, Design, Sample, and Study Country

Participants across the included studies were primarily adults recovering from COVID-19 with persistent symptoms, typically assessed weeks to months post-infection. Most were aged between their mid-30s and mid-60s, with a slight predominance of female participants. The review encompassed 23 observational studies, ranging from cross-sectional to prospective cohort and mixed-methods designs, with sample sizes varying from fewer than 50 to over 68,000. The remaining 27 were interventional, including 17 randomized controlled trials. Altogether, the 50 studies spanned 24 countries, with the largest contribution from Italy (*n* = 13), followed by Serbia, the United States, the United Kingdom, and Spain (*n* = 4 each), and various others represented by one or two studies.

### 3.3. Long COVID-19: Terminology and Definitions Across Studies

Studies used a range of terms—post-acute sequelae of COVID-19 (PASC), post-COVID-19 syndrome, chronic COVID-19, prolonged COVID-19, long-haul COVID-19, persistent symptoms, and COVID-19 survivors—often interchangeably to describe prolonged symptoms following acute SARS-CoV-2 infection. However, definitions and assessment criteria varied considerably across studies, reflecting both the evolving understanding of the condition and the absence of a standardized diagnostic framework during the study periods.

The duration and definition of long COVID-19 varied widely, ranging from symptoms persisting for more than three weeks to criteria requiring symptoms lasting ≥12 weeks or even beyond six months. Several studies followed WHO or NICE guidelines, which define long COVID-19 as symptoms that begin or continue ≥12 weeks post-infection and are not explained by other conditions. Other studies identified cases using clinical judgment, ICD-10 code U09, or recruitment from post-COVID-19 clinics. In community and outpatient settings, long COVID-19 was often defined by self-reported symptoms—such as fatigue or cognitive dysfunction—lasting 30 to 90 days or more after diagnosis.

### 3.4. Strength of Evidence

Of the 50 studies reviewed, 11 (22%) were rated as high strength, reflecting randomized controlled trials or large observational studies with robust methodology. Almost half of the studies (N = 24, 48%) were rated moderate and 15 studies (30%) were rated low strength (*n* = 12) or low–moderate (*n* = 3), characterized by small sample sizes, cross-sectional, or open-label designs. Overall, 38 studies (76%) reported a significant association between nutrition or dietary exposure and long COVID-19 or its symptoms.

### 3.5. Main Study Outcomes

Fatigue was the most investigated symptom, assessed in 20 of 50 studies (40%) using validated tools such as the Chalder Fatigue Questionnaire, Fatigue Severity Scale (FSS), and PROMIS, mostly through self-report. Physical performance was evaluated in 18 studies using objective measures such as the Six-Minute Walk Test (6MWT), handgrip strength, and chair stand tests, as well as self-reported function and measures from the SARC-F tool (Strength, Assistance with walking, Rise from a chair, Climb stairs, and Falls) to assess sarcopenia and muscle weakness. Biomarkers were also analyzed, focusing on inflammation (e.g., CRP, IL-6), oxidative stress, and gut microbiota. Long COVID-19 symptom clusters—including cough, dyspnea, anosmia, and headache—were assessed via symptom inventories or composite scores. Mental health, quality of life (QoL), well-being, and sleep were examined in 16 studies.

### 3.6. Results of Individual Sources of Evidence

**Figure 2:**
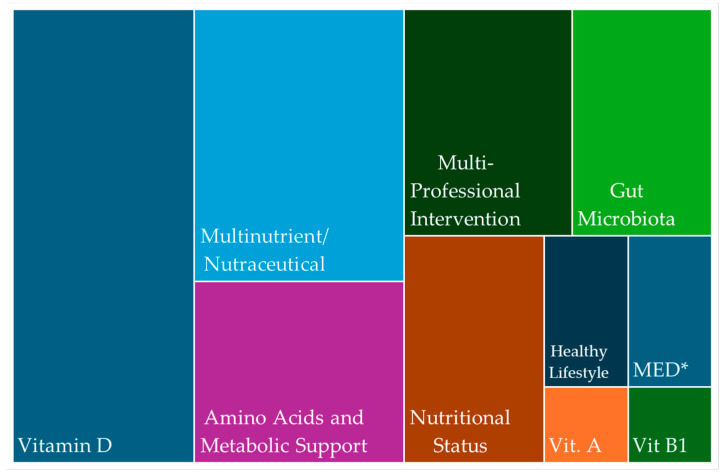
Distribution of dietary and nutritional exposures assessed in studies on long COVID-19 in adults. * MED, the Mediterranean diet; Vit, vitamin.

Of the 50 studies, 13 focused on vitamin D. Other vitamins examined included C, A, B1, and K. Zinc and magnesium were commonly assessed, often with other nutrients. Nine studies investigated multinutrient or nutraceutical supplements, while six evaluated amino acids or metabolic compounds such as L-arginine, creatine, and oxaloacetate. Six studies targeted gut microbiota using probiotics, synbiotics, paraprobiotics, or dietary strategies such as beetroot juice and the Mediterranean diet. Two large cohort studies analyzed pre-infection healthy lifestyle factors. Five studies assessed nutritional status in post-hospitalized or ICU patients. Seven implemented multicomponent lifestyle interventions integrating diet, exercise, and psychosocial support (Figure 2).

This treemap visualization displays the relative frequency of exposure categories assessed across the included studies. Larger blocks represent more commonly studied categories, such as vitamin D, nutraceutical/multinutrient formulations, and amino acid/metabolic support. Less frequently studied categories include individual micronutrients (e.g., vitamins A and B1), the Mediterranean diet, and healthy lifestyle patterns. The “Multi-Professional Intervention” category refers to interventions that combine dietary components with physical or psychological therapies. Gut microbiota-targeted approaches.

#### 3.6.1. Vitamin D

Overall, 13 studies examined vitamin D—10 as a primary exposure and three in combination with other nutrients. Seven of these studies reported significant findings [32,34,36,37,38,39,42] (Table 2). For example, Di Filippo et al. evaluated 100 COVID-19 survivors (50 with long COVID-19, 50 without) six months after hospitalization, applying the NICE criteria for long COVID-19 (≥2 persistent symptoms without an alternative diagnosis) [34]. Those with long COVID-19 had significantly lower vitamin D levels than those without (20.1 vs. 23.2 ng/mL, *p* = 0.03). Chadda et al. [39] studied 392 NHS healthcare workers who had self-isolated due to COVID-19 and found that VDD was associated with both a greater number and longer duration of COVID-19 symptoms, particularly fatigue and body aches.

In a cross-sectional study from northeastern Taiwan, Chen et al. [36] assessed serum zinc and vitamin D levels in long COVID-19 outpatients, finding zinc deficiency in 27.3% and vitamin D deficiency in 29% of participants. Among those with low zinc, 63.6% had serum concentrations below 700 µg/L (mean: 603.3 ± 66.3 µg/L), while the mean vitamin D level among deficient individuals was 14.6 ± 2.7 ng/mL. A significant positive correlation between zinc and vitamin D levels (*p* < 0.05) suggested interrelated micronutrient deficiencies.

Three clinical trials reported significant findings. Charoenporn et al. [38] conducted an 8-week, double-blind, randomized, placebo-controlled trial in 80 individuals with post-COVID-19 syndrome, administering 60,000 IU of vitamin D weekly. The intervention showed significant improvements in fatigue (*p* = 0.024), anxiety (*p* = 0.011), and cognitive function (*p* = 0.012), although no effects were observed for depression, sleep quality, or inflammatory markers. Vitamins K2 and D3, known for their synergistic anti-inflammatory and vasoprotective effects, were evaluated in a randomized trial involving 151 adults [42]. Over 24 weeks, it was found that combined K2/D3 supplementation significantly reduced symptoms, improved markers of inflammation and oxidative stress (e.g., sTNF-RI, sCD163, oxLDL), and decreased fungal translocation (β-D-glucan). The intervention also enhanced gut barrier integrity, suggesting that K2/D3 may alleviate symptoms by modulating systemic inflammation and gut permeability. In another trial [37], combined magnesium and vitamin D supplementation in long COVID-19 patients significantly improved depression, with 73.2% achieving remission compared to 34.5% in the vitamin D-only group (*p* = 0.006).

By contrast, six observational studies [30,31,35,40,41] and one randomized controlled trial [33] reported no significant associations (Table 2). All observational studies assessed vitamin D status via serum biomarkers. Townsend et al. found no association between vitamin D levels and fatigue or exercise tolerance in 149 patients [31]. Kaddah et al. similarly reported no link between vitamin D levels and symptom burden in 84 patients, despite widespread deficiency [40]. Hikmet et al. found no relationship between vitamin D and symptom severity among 442 patients attending a Danish post-COVID-19 clinic [35]. Likewise, in a retrospective analysis of 8300 vitamin D-deficient individuals and matched controls, Wu et al. found no association with long COVID-19 outcomes [41]. The CovILD prospective cohort (N = 109) also reported no associations between 25(OH)D levels—measured at diagnosis and at 8-week follow-up—and persistent symptoms, impaired lung function, inflammatory markers, or CT abnormalities, despite high rates of deficiency [30]. Finally, a multicenter RCT of 144 hospitalized patients found no benefit of a single 200,000 IU dose of vitamin D_3_ on persistent symptoms or quality of life at 6- and 12-months post-discharge [33].

Vitamin A was examined in a pilot study targeting olfactory dysfunction [43], where oral supplementation combined with olfactory training improved both smell function and neural activity in the olfactory network—potentially supporting appetite and reducing fatigue related to long COVID-19. Vitamin B1 at 600 mg/day significantly improved fatigue, myalgia, anosmia, and sleep issues in long COVID-19 in an open-label RCT [44].

#### 3.6.2. Multinutrient, Nutraceutical, and Combined Formulations

Multinutrient and nutraceutical formulations have been explored for their potential to alleviate fatigue, inflammation, physical impairment, and cognitive dysfunction caused by COVID-19 [45,46,47,48,49,50,51,52,53] (Table 3). Multinutrient supplements include essential vitamins, minerals, and amino acids aimed at restoring nutritional balance and supporting immune and metabolic function. Nutraceuticals often feature bioactive compounds like plant extracts, polyphenols, probiotics, or fermented ingredients with antioxidant, anti-inflammatory, or immunomodulatory effects. Apportal^®^ (PharmaNutra Spa, Pisa, Italy), a combined formula with 19 nutrients—B vitamins, Sucrosomial^®^ (PharmaNutra Spa, Pisa, Italy), minerals, amino acids, and adaptogenic plant extracts—was evaluated in two studies. One reported reduced fatigue and improved quality of life in 76.6% of participants by day 14 and 90% by day 28 [45]; the other study noted gains in strength, endurance, and self-rated health [48].

A pilot case–control study in Italy evaluated a multicomponent supplement containing essential amino acids and Krebs cycle intermediates in 66 COVID-19 survivors; after 8 weeks, the intervention group showed significant improvements in muscle mass, physical performance (e.g., handgrip, sit-to-stand, walking test), and quality of life compared to controls [47]. Similarly, another pilot observational study found that a supplement combining acetyl-L-carnitine, hydroxytyrosol, and vitamins B, C, and D significantly improved self-perceived energy (↑123%) and reduced tiredness and tension (~50%) after 15 days, compared to no change in non-users [46].

PIRV-F20^®^ (Farmagens Health Care s.r.l., Naples, Italy), a multicomponent supplement with vitamins, trace elements, probiotics, lactoferrin, lysozyme, and resveratrol, was tested in 44 long COVID-19 patients with reduced exercise capacity. After six weeks, the supplement group improved more in six-minute walking distance (+40 m vs. +10 m, *p* = 0.02) than controls, though no difference was seen in strength or symptoms [53]. In a randomized, placebo-controlled study, Kharaeva et al. [49] evaluated fermented Carica papaya and *Morinda citrifolia* (Noni) in 188 post-COVID-19 patients (mean age 48.7; 64.8% female). Taken twice daily for 20 days, the supplement significantly reduced fatigue (61.5% vs. 24.3%, *p* < 0.001), cognitive symptoms (59.4% vs. 21.6%, *p* < 0.001), and joint pain (66.2% vs. 33.8%, *p* = 0.002), and decreased IL-6, TNF-α, and oxidative stress markers. In a multicenter randomized trial in Spain, 246 COVID-19 patients received a 14-day micronutrient formula at illness onset. Despite correcting deficiencies, there was no difference in long COVID-19 prevalence at 180 days (27.7% vs. 25.0%, *p* = 0.785) [51]. A 2-month placebo-controlled trial tested an oral food supplement (OFS) with Echinacea, rosehip, propolis, royal jelly, zinc, and vitamin C. The intervention group had lower inflammatory markers (CRP, NLR, MLR), improved vitamin D, and less fatigue and better quality of life [52].

#### 3.6.3. Amino Acids and Metabolic Support

L-arginine is a conditionally essential amino acid involved in protein synthesis, nitric oxide production, and immune function. Altered arginine metabolism in COVID-19 has been associated with immune and vascular dysfunction, prompting interest in its therapeutic potential for long COVID-19 [54,55,56,57,58,59] (Table 4). A randomized controlled trial assessed the effects of twice-daily L-arginine (1.66 g) plus liposomal vitamin C (500 mg) for 28 days in long COVID-19 patients. Supplementation significantly improved serum L-arginine concentrations and L-arginine/ADMA ratios, suggesting enhanced nitric oxide bioavailability and vascular function [54]. In the LINCOLN nationwide survey, 1390 long COVID-19 patients received either L-arginine with vitamin C or a multivitamin formulation. After 30 days, the L-arginine group reported significantly lower symptom scores compared to the multivitamin group [56].

Another study of 505 patients compared two arginine-based formulations—Astenor Energy^®^ (Biessen Pharma, Romania) and Astenor Forte^®^ (Biessen Pharma, Romania)—prescribed in routine care. After three months, both groups showed significant reductions in fatigue, with 68.7% of participants reporting no fatigue, compared to 27.7% at baseline [57].

Creatine, synthesized from arginine and glycine, plays a key role in cellular energy production and recovery. Two clinical trials examined its use in long COVID-19 patients. In an 8-week double-blind study, 15 participants were randomized to receive creatine (8 g), creatine plus glucose (8 g + 3 g), or placebo. Supplementation increased brain creatine levels and significantly improved long COVID-19 symptoms such as fatigue, headaches, and cognitive issues [59]. A separate 6-month randomized trial in 12 patients demonstrated that 4 g daily creatine significantly increased creatine levels in muscle and brain tissue and led to reductions in fatigue and other long COVID-19 symptoms, including ageusia and breathing difficulties [58].

Oxaloacetate, a cellular energy metabolite, has been proposed to counteract metabolic dysfunction contributing to fatigue. A six-week, open-label trial evaluated anhydrous enol-oxaloacetate in 76 ME/CFS patients and 43 long COVID-19 patients. Doses ranged from 500 mg to 1000 mg (up to three times daily). Fatigue scores, measured by the Chalder Fatigue Questionnaire, improved significantly across groups, with dose-dependent reductions. Among long COVID-19 patients, fatigue improved by up to 46.8% [55].

#### 3.6.4. Gut Microbiota Modulation in Long COVID-19

Long COVID-19 patients often exhibit gut microbiota dysbiosis, potentially contributing to persistent symptoms such as gastrointestinal discomfort, fatigue, cognitive impairment, and mood disturbances. Accordingly, microbiome-targeted interventions—including probiotics, synbiotics, paraprobiotics, and dietary strategies—are emerging as potential therapies [60,61,62,63,64,65] (Table 5). Supporting this approach, the UK Phyto-V Study, a randomized, double-blind, placebo-controlled trial, tested a phytochemical-rich nutritional capsule plus probiotic in 147 symptomatic COVID-19 patients. The intervention group showed greater reductions in fatigue and cough and improved well-being, particularly among those with prolonged symptoms, older age, sedentary lifestyle, prior hospitalization, and gastrointestinal issues [60].

Docampo et al. piloted a four-week paraprobiotic intervention in six long COVID-19 patients, observing improvements in fatigue, dysautonomia, and depression based on self-reports, wearable data, and immunophenotyping. Immune activation markers were also reduced, suggesting immunomodulatory effects [61].

In a large randomized, placebo-controlled trial, Lau et al. [62] evaluated the synbiotic SIM01 (comprising Bifidobacteria and prebiotics) in 463 individuals with post-acute COVID-19 syndrome (long COVID-19). After six months, participants receiving SIM01 reported significant alleviation of core long COVID-19 symptoms—including fatigue, memory loss, brain fog (difficulty concentrating), gastrointestinal disturbances, and general unwellness (all *p* < 0.01). These improvements were accompanied by enhanced gut microbial diversity and enrichment of beneficial bacteria such as B. adolescentis and F. prausnitzii, known for their roles in short-chain fatty acid production and immune regulation. SIM01 also led to reductions in antimicrobial resistance genes and suppressed pathways like the urea cycle, which has been implicated in post-viral fatigue.

Other strategies include Calvani et al.’s [63], who, in a 14-day RCT with 31 patients, administered nitrate-rich beetroot juice. Although between-group differences were not significant, the beetroot group showed improved fatigue resistance, walking distance, and increases in beneficial gut bacteria (Akkermansia, Roseburia) and fecal metabolites, along with shifts in cytokines like interferon-gamma and MIP-1β.

Diet may also modulate the gut–immune axis. Cuevas-Sierra et al. [64] found that high adherence to the Mediterranean diet correlated with lower LDL, glucose, and LDH levels and beneficial microbiota shifts. Extra virgin olive oil intake was associated with anti-inflammatory microbiome-mediated effects. Finally, Ranisavljev et al. [65] conducted a three-month RCT in 26 patients with post-COVID-19 ME/CFS using a synbiotic containing probiotics, prebiotics, and zinc. Both groups improved in fatigue, but the intervention group had greater reductions in post-exertional malaise and increased brain choline and creatine levels in regions tied to fatigue and cognition.

#### 3.6.5. Pre-Infection Lifestyle and Risk of Long COVID-19

In addition to efforts focused on managing symptoms and supporting recovery, other studies suggest that lifestyle behaviors prior to infection—especially dietary habits—may play a protective role in reducing the likelihood of developing long COVID-19 [66,67] (Table 6). A prospective cohort study within the Nurses’ Health Study II evaluated 1981 U.S. women who tested positive for SARS-CoV-2 between April 2020 and November 2021. Adherence to five or six out of six healthy behaviors—maintaining a healthy BMI, never smoking, engaging in regular physical activity, consuming a high-quality diet, moderate alcohol intake, and sufficient sleep—was associated with a 49% lower risk of a post-COVID-19 condition (PCC) (RR = 0.51, 95% CI: 0.33–0.78). Among these, healthy body weight and adequate sleep emerged as independent protective factors. If causal, up to 36% of PCC cases could be preventable through modifiable lifestyle improvements [66].

Supporting this, in a large prospective study using UK Biobank data (*n* = 68,896), males and females similarly found that adherence to 6–10 healthy behaviors—including a diet high in fruits and vegetables, adequate oily fish intake, and low processed meat consumption—was associated with a 36% lower risk of developing multisystem post-COVID-19 sequelae (HR = 0.64, 95% CI: 0.58–0.69). These protective associations extended across fatigue, cardiometabolic, neurological, and respiratory symptoms, and remained robust across acute and post-acute phases, independent of comorbidities [67].

#### 3.6.6. Prolonged Nutritional Status After COVID-19

Five studies examined the prolonged nutritional and functional consequences of COVID-19 among patients who experienced severe or extended illness [68,69,70,71,72]. In the NUTRICOVID study, Alvarez-Hernandez et al. [69] followed 199 ICU survivors in Spain (mean age 61.3 ± 11.6 years) and found that 76% were at nutritional risk at hospital discharge based on the Malnutrition Universal Screening Tool (MUST). One year later, 35% remained at risk, and approximately 25% showed signs of sarcopenia. Despite receiving nutritional support—including oral, enteral, or parenteral nutrition—only 36% met at least 75% of their energy needs and 23% met protein targets at discharge. At one year, 43% still failed to meet energy needs and 63% remained below protein goals (Table 6).

Similarly, Gérard et al. [68] investigated outcomes in a French cohort of 288 post-hospitalized patients (mean age 59.8 ± 16.6) due to COVID-19, finding that 36% remained malnourished six months after discharge. Fifteen percent reported persistent muscle weakness and reduced physical performance, with obesity and ICU admission predicting worse outcomes.

Lakenman et al. [70] extended this line of evidence by following 48 ICU survivors in the Netherlands for one year. Although most participants regained their pre-illness weight and physical performance—achieving 92% of baseline handgrip strength and 95% of six-minute walk capacity—body composition remained altered. Half of the participants had an elevated fat mass index, and 19% had a low fat-free mass index. Median nutritional intake reached only 90% of resting energy expenditure and 77% of protein needs.

In Mexico, Mejía Alonso et al. [72] evaluated 66 COVID-19 pneumonia survivors (mean age 51.3 ± 14.7) referred for rehabilitation. Over time, participants showed improvements in protein deficiency, muscle thickness, handgrip strength, respiratory function, and quality of life. However, delayed referral and excess weight were associated with poorer six-minute walk performance.

Finally, Muzaffar et al. [71] explored post-COVID-19 nutrition and lifestyle in a younger Malaysian cohort, comparing 54 recovered individuals (aged 18–30) with 54 healthy controls. While sleep quality and weight change did not differ significantly between groups, the recovered individuals exhibited significantly lower dietary diversity. Notably, neither diet diversity nor sleep quality was correlated with weight outcomes.

#### 3.6.7. Multi-Professional Intervention and Diet Modification

Several recent studies have examined dietary modifications integrated within multi-professional interventions for post-COVID-19 recovery, reporting comparable findings across diverse populations and care settings [19,73,74,75,76,77,78] (Table 6). In Serbia, a prospective cross-sectional study evaluated 76 working-age adults (mean age 30.6 ± 1.5 years) within six months of PCR-confirmed infection. Although participants reported reduced physical activity—particularly aerobic and high-intensity exercise—and persistent symptoms such as fatigue (23.7%), dietary patterns remained largely stable aside from an increase in water intake (*p* = 0.049) [74].

Building on this, several interventional studies in Europe and Latin America have demonstrated the potential benefits of structured nutrition and rehabilitation programs. In Portugal, Sousa-Catita et al. assessed 118 older adults (mean age ~72) enrolled in a 30-day multidisciplinary program that included physiotherapy, speech therapy, and individualized nutrition following ESPEN guidelines. Nutritional support—ranging from dietary counseling to oral supplementation—led to improvements in BMI, handgrip strength, and MNA^®^ scores, particularly among ICU survivors [73].

Complementing these findings, Ryal et al. in Brazil implemented an 8-week lifestyle program for 55 overweight or obese COVID-19 survivors (mean age 49.9 ± 13.1), which included psychoeducation, physical activity, and group-based nutritional education. Participants experienced significant improvements in psychological well-being, anxiety, depression, and quality of life [76]. In a companion study, Sordi et al. reported physical benefits such as reductions in fat mass (−0.6 kg), increases in lean mass (+0.8 kg), enhanced handgrip strength, and smaller waist circumference—outcomes relevant to post-COVID-19 fatigue and functional recovery [75].

In Germany, Pink et al. conducted a secondary analysis of a 12-week RCT involving 46 individuals with post-COVID-19 fatigue (median age 47; 74% female). Participants completed 7-day food diaries and wore activity/sleep trackers. Compared to controls, the post-COVID-19 group demonstrated healthier eating habits, including greater adherence to the Mediterranean diet and higher intake of omega-3s and vitamin C. However, no clear associations emerged between nutrient intake and fatigue severity, reflecting the complexity of nutritional interventions in this context [77].

A larger randomized controlled trial in the UK—the ReDIRECT study—evaluated a remotely delivered weight-loss program in 234 adults with long COVID-19 (mean age ~46; 85% female; BMI >27 kg/m^2^, or >25 for South Asians). The intervention began with 12 weeks of total diet replacement (850 kcal/day) and led to significantly greater improvement in participants’ dominant long COVID-19 symptoms compared to controls (mean difference −0.34; 95% CI −0.67 to −0.01) [78].

Adding to this international evidence base, the BioICOPER study in Spain assessed 305 adults with long COVID-19 and found that higher adherence to the Mediterranean diet was significantly associated with lower BMI, waist circumference, uric acid levels, and number of metabolic syndrome components, as well as higher HDL cholesterol. These findings suggest that greater adherence to an anti-inflammatory dietary pattern may help mitigate cardiometabolic dysfunction commonly observed in individuals with long COVID-19, underscoring the relevance of nutrition in post-COVID-19 recovery strategies [19].

### 3.7. Summary of Evidence on Key Nutritional Exposures in Long COVID-19

Table 7 summarizes the evidence across various nutritional exposures. Studies on amino acids/metabolic support (100% of six studies), multinutrient/nutraceutical formulations (89% of nine studies), gut microbiota-targeted therapies (83% of six studies), and multicomponent lifestyle interventions (71% of seven studies) reported the highest proportions of significant findings. Vitamin D was the most frequently studied intervention (10 studies), with 40% reporting significant effects when examined as a primary exposure and 100% when provided in combination with other nutrients such as zinc, magnesium, or vitamin K2. While no formal critical appraisal was conducted, several categories—including vitamin D, amino acids, multinutrient formulations, and gut microbiota-targeted therapies—included randomized controlled trials, suggesting a foundation for future high-quality research.

## 4. Discussion

This scoping review offers a comprehensive synthesis of global evidence on the role of diet and nutrition in the management and recovery of long COVID-19. Across 50 studies, findings span a wide array of nutritional exposures, including individual micronutrients (e.g., vitamins D, K_2_, A, B1, zinc, magnesium), amino acid-based (e.g., L-arginine, creatine) and multinutrient formulations, phytochemicals, microbiota-targeted therapies (probiotics, synbiotics), and whole-diet patterns such as the Mediterranean diet. Several diet modifications were nested within multi-professional frameworks, integrating dietary support with physical rehabilitation and psychological care, thereby offering real-world insights into feasible therapeutic strategies. This diversity reflects the condition’s varied symptomatology and underlying immune, metabolic, and vascular disruptions, indicating the need for multifaceted nutritional strategies in long COVID-19 recovery and management.

Notably, nearly 76% of the included studies reported significant clinical benefits in mitigating long COVID-19-related symptoms, highlighting nutrition’s role as both a modulator of pathophysiology and a viable adjunct to recovery. Given the condition’s complexity, several plausible biological mechanisms—such as immune modulation, mitochondrial support, and gut–brain axis restoration—may underlie the observed nutritional effects. Mechanistic evidence was particularly robust for vitamin D supplementation. Three randomized controlled trials demonstrated that higher-dose vitamin D supplementation, alone or in combination with micronutrients such as vitamin K_2_, improved fatigue, anxiety, cognitive symptoms, or depression [37,38,42]. These effects are thought to result from vitamin D’s regulation of neurotransmitter synthesis, preservation of blood–brain barrier integrity, and modulation of monocyte-driven inflammation and gut permeability—supporting a multi-system model of neuroimmune recovery [19,79,80,81]. However, not all trials have yielded consistent results. A large post hoc analysis of a randomized trial found no benefit of a single 200,000 IU dose of vitamin D_3_ on persistent symptoms or quality of life at one year after COVID-19 hospitalization [30]. As this study was conducted early in the pandemic, before variant evolution and vaccination, and relied on telephone follow-up with limited symptom detail, its findings may not fully reflect the broader, evolving spectrum of long COVID-19 presentations.

Evidence also supports the role of amino acid-based interventions. L-arginine, particularly when combined with liposomal vitamin C, yielded consistent improvements in walking capacity, muscle strength, endothelial function, or fatigue across trials [51,53]. Mechanistically, L-arginine enhances nitric oxide (NO) bioavailability, improving vascular dilation and oxygen delivery, while vitamin C stabilizes NO and reduces oxidative stress. These pathways directly address endothelial dysfunction and fatigue—two hallmark features of long COVID-19—while also supporting mitochondrial and muscular recovery [80].

Beyond individual nutrients, several other studies in this review demonstrated the potential of microbiota-targeted interventions. The RECOVERY trial, a double-blind RCT, showed that a synbiotic formula (SIM01) significantly alleviated fatigue, memory loss, gastrointestinal (GI) disturbances, and general unwellness [62]. These benefits were accompanied by increased microbial diversity, enriched short-chain fatty acids (SCFAs)-producing bacteria (e.g., B. adolescentis, F. prausnitzii), and suppression of urea cycle and antibiotic resistance pathways. These compositional and functional microbiome shifts were correlated with symptom resolution, further reinforcing the centrality of the gut–immune–brain axis in long COVID-19. Similar findings were reported in other studies using probiotics, paraprobiotics, and adherence to Mediterranean dietary patterns [60,61,63,64,65,81,82].

While these interventions focus on supporting recovery, complementary evidence from this review suggests that nutritional status prior to infection may also influence susceptibility to long COVID-19 and the severity of post-acute outcomes. Two large prospective cohorts found that individuals with healthy lifestyle behaviors—including optimal BMI and high-quality dietary patterns—had a lower risk of developing long COVID-19 or experiencing severe sequelae [66,67]. Among those already affected, studies revealed that both acute-phase and post-acute nutritional status significantly shaped recovery trajectories. Protein-energy supplementation improved strength and function, reduced malnutrition, and mitigated catabolic muscle loss, particularly among ICU survivors [68,69,70,71,72].

Although nutrition was not evaluated in isolation in all studies, real-world interventions that incorporated dietary modification alongside physical and psychological support showed compelling benefits. One structured dietary weight-loss program in individuals with overweight or obesity demonstrated significant improvements in long COVID-19 symptom burden, lending further credence to the hypothesis that nutritional strategies can shape systemic recovery [78]. Interventions aligned with ESPEN recommendations and Mediterranean dietary principles similarly emphasized the importance of diet quality, energy balance, and anti-inflammatory food patterns in modulating long-term outcomes [19,73,74,75,76,77,78].

As a body of evidence, this scoping review positions diet and nutrition as underrecognized yet biologically grounded tools in the management of long COVID-19. Mechanisms such as immune modulation, vascular repair, neuroprotection, and microbial balance underpin the observed benefits. Nutritional strategies—particularly those rich in anti-inflammatory, antioxidant, and amino acid-based components, and/or targeting the gut microbiota—may help alleviate persistent symptoms. Including such approaches within coordinated, multi-disciplinary care could play a pivotal role in restoring resilience and supporting long-term recovery [79,80,81,82,83,84,85,86,87].

From a clinical perspective, the findings underscore the need for multifaceted nutritional strategies to manage long COVID-19. Effective approaches include individualized counseling, screening for nutritional deficiencies (e.g., vitamin D, protein), and aligning dietary goals with established frameworks such as the ESPEN guidelines, USDA MyPlate, or the Dietary Guidelines for Americans (DGA). Diets emphasizing anti-inflammatory and microbiota-supportive components—such as fruits, vegetables, whole grains, legumes, fatty fish, and unsaturated fats like extra virgin olive oil—have systemic benefits, enhancing immune function and promoting gut microbiome diversity.

Clinical trials further support the use of supplements such as synbiotics (e.g., SIM01) and L-arginine (~2 g/day), especially when combined with vitamin C. For individuals recovering from hospitalization or experiencing malnutrition, adequate protein intake (1.2–1.5 g/kg/day) and energy-dense food fortification are essential to restore muscle mass and physical performance. Vitamin D supplementation—particularly in combination with vitamin K_2_—has also shown efficacy in alleviating core long COVID-19 symptoms. In contrast, diets high in ultra-processed foods, added sugars, and pro-inflammatory fats may worsen immune dysregulation and hinder recovery. Taken together, these nutritional strategies offer accessible, non-pharmacologic tools that can be feasibly implemented across clinical and community settings to support long-term recovery in individuals affected by long COVID-19.

However, several limitations should be acknowledged. While this review incorporated a structured methodology and broad database coverage, it was a scoping review—not a systematic review—and as such, no formal quality appraisal was performed, and causal inferences cannot be made. Without a risk of bias assessment or advanced synthesis methods, conclusions about effectiveness or other outcomes cannot be drawn. Although full-text screening was conducted independently by two reviewers, title and abstract screening was performed twice by a single reviewer approximately six months apart. This approach may have introduced some selection bias; however, the use of predefined eligibility criteria and a structured screening process helped promote consistency. Repeating the screening at two time points served as an internal check to reduce the risk of omitting relevant studies and provided a pragmatic alternative when dual screening was not feasible [88]. We also did not involve a third reviewer to resolve discrepancies during full-text screening, which may limit objectivity in rare cases of disagreement; however, all discrepancies were resolved through discussion and consensus between the two reviewers. Considerable heterogeneity in study design, population characteristics, outcome measures, and definitions of long COVID-19 further limits comparability across studies. Moreover, sample sizes were often small, follow-up durations short, and many studies lacked adequate control conditions or placebo blinding. Few studies adequately adjusted for confounders such as comorbidities, vaccination status, or viral variants. Lastly, relevant non-English or gray literature may have been missed despite the comprehensive search strategy.

Building on the current evidence base from this scoping review, future research should prioritize systematic reviews focused on specific nutrients or dietary patterns to address clearly defined research questions related to distinct long COVID-19 symptoms or symptom clusters. Looking forward, well-powered, mechanistically informed randomized controlled trials are urgently needed to assess the efficacy of dietary interventions for long COVID-19 across key symptom clusters such as fatigue, cognitive dysfunction, and gastrointestinal distress. Trials incorporating biomarker endpoints, microbiome profiling, and functional outcomes will be critical to advancing personalized nutrition strategies. In parallel, observational cohorts should continue to explore the protective role of baseline dietary patterns in shaping long-term COVID-19 risk and recovery trajectories.

## 5. Conclusions

Diet and nutrition should be more prominently recognized in long COVID-19 care—not only as supportive measures but as active components of recovery strategies. This review points to their potential to address key symptoms through biologically plausible mechanisms yet also highlights the imperative need for rigorous trials to confirm efficacy. Moving forward, integrating personalized, evidence-based nutritional guidance into multidisciplinary care may offer a low-risk, scalable approach to improve recovery outcomes and enhance resilience in individuals affected by long COVID-19.

## Figures and Tables

**Table 1:** Eligibility criteria.

Inclusion	Exclusion
Peer-reviewed journal articles.	Commentaries, case reports, letters, books, dissertations, editorials, conference proceedings.
Participants must have long COVID-19 symptoms or a history of post-COVID-19 recovery.	Long COVID-19 or post-COVID-19 symptoms were not under investigation.
Measurable outcomes related to post-COVID-19 recovery/symptoms.	
Human adults (≥18 years old).	Children
Published in English.	Not published in English.
Quantity study designs, such as RCTs, cohort, case–control, or observational studies.	Qualitative, non-quantitative, or review study.
Publication date from 2020 onward to ensure relevance to long COVID-19.	Studies published before 2020.
Observational or Intervention studies focused on nutrition, diet, or nutritional supplements.	Interventions involving rehabilitation programs without dietary components.

**Table 2:** Summary of studies examining vitamins, D, B1, and A in the context of long COVID-19: study characteristics and key findings.

Study, Year, Ref.	Country	Study Design	Study Sample	Long COVID-19 Assessment	Type of Nutritional Exposure	Outcomes Assessed	Assessment Tools/Criteria	Main Results	Significance and Strength of Evidence
Pizzini et al., 2020 [30]	Austria	Prospective CovILD study	N = 109 Age: 58 ± 14 y 40%, female.	CovILD registry 8 weeks after hospitalization/ outpatients with COVID-19 symptoms	Biomarker assessment: serum 25(OH)D levels	Parathyroid hormone (PTH), inflammatory markers, lung function, and CT findings.	Standard pulmonary function tests, VDD (25(OH)D < 30 nmol/L) CRP, IL-6	VDD is frequent among COVID-19 patients but not associated with disease outcomes after eight weeks	Non-sig; Moderate
Townsend et al., 2021 [31]	Ireland	Cross- sectional	N = 149 Age 48.0 ± 15.0 y 59.1% female.	Post-COVID-19 outpatient symptoms at a median of 79 days post-infection	Biomarker assessment: serum 25(OH)D levels	Fatigue; exercise tolerance; inflammation	CFS, 6MWT, modified Borg scale; CRP, IL-6	No significant association between serum vitamin D levels and fatigue or exercise tolerance.	Non-sig; Moderate
Galluzzo et al., 2022 [32]	Italy	Cross- sectional	N = 681. Age 53.4 ± 15.2 y 49% female	COVID-19 survivors admitted to the post-COVID-19 outpatient setting	Biomarker assessment: serum 25(OH)D levels	Physical performance	6MWT, One-minute chair stand, Handgrip strength	VDD was associated with reduced physical performance; participants with normal levels walked farther on the 6MWT (475.0 m vs. 421.9 m; *p* < 0.01).	Sig; Moderate
Fernandes et al., 2022 [33]	Brazil	Double-blind, placebo-controlled, RCT	N = 144 Age: 54.3 ± 13.1 y 47.9%, female	Hospitalized patients with moderate to severe COVID-19 that were followed-up for 1 year	Supplement intervention: a single high dose of vitamin D_3_ (200,000 IU)	Persistent or new symptoms (e.g., fatigue, joint pain, myalgia)	Symptoms at 6 and 12 months after discharge QoL (SF-36)	No significant differences between groups were observed for fever, cough, fatigue, fever, myalgia, joint pain, QoL, and new or persistent symptoms up to 1-year of follow-up	Non-sig; Moderate
Di Filippo et al., 2023 [34]	Italy	Cross- sectional Retrospective	N = 100 Age: 61 (51–70) y 44% female	National Institute for Health and Care Excellence (NICE) *n* = 50 long COVID-19 vs. *n* = 50 without	Biomarker assessment: serum 25(OH)D levels	Long COVID-19 diagnosis	NICE case definition	Lower vitamin D levels at follow-up were associated with long COVID-19 (20.1 vs. 23.2 ng/mL; *p* = 0.03; OR = 1.09, 95% CI: 1.01–1.16; *p* = 0.008).	Sig; Moderate
Hikmet et al., 2023 [35]	Denmark	Cohort study	N = 442 Age 47 ± 12.7 y 72% female	long COVID-19 at specialized post-COVID-19 clinic; symptoms > 12 weeks	Biomarker classification: serum 25(OH)D3 levels (cutoff: <50 nmol/L vs. normal)	Symptom burden	PCQ (31-symptom sum score)	No significant differences in symptom prevalence or severity between vitamin D-deficient and -sufficient groups.	Non-sig; Moderate
Chen et al., 2023 [36]	Taiwan	Retrospective case	N = 55 Age 49.3 ± 17.5 y 61.8% female	Symptoms persisted for more than 42 days	Biomarker assessment: serum vitamin D and zinc levels	Duration of long COVID-19 symptoms; inflammation	Self-report (symptom duration); inflammatory biomarkers	Vitamin D (29.1%) and zinc (27.3%) deficiencies were associated with long COVID-19 (*p* < 0.05); zinc deficiency linked to elevated fibrinogen, and VDD to delayed recovery.	Sig; Low
Rodríguez-Morán et al., 2024 [37]	Mexico	RCT Open-label	N = 60 Age 52.8 ± 12.6 y	Hypomagnesemia, VDD, and MMD related to long COVID-19	Supplement intervention: Magnesium chloride (1300 mg/day) + vitamin D (4000 IU/day) vs. vitamin D alone for 4 months	Depressive symptoms MMD	MMD defined as BDI score 11–30; improvement defined as BDI <11	Depressive symptoms improved more in the intervention group (BDI 28.8 → 9.2; *p* < 0.01) than in controls (28.4 → 21.6; *p* < 0.05); 73.2% vs. 34.5% achieved BDI <11 (*p* = 0.006).	Sig; Moderate
Charoenporn et al., 2024 [38]	Thailand	Double-blind RCT	N = 80 Age 34.1 ± 10.5 y 75%, female	at least one post-symptom within 3 months of COVID-19 onset and lasting for >1 month.	Supplement intervention: 60,000 IU/week of vitamin D2 vs. placebo for 8 weeks	Fatigue, anxiety, depression, sleep, cognition, inflammation	Questionnaires; IL-6, CRP	Vitamin D supplementation improved fatigue (*p* = 0.024), anxiety (*p* = 0.011), and cognition (*p* = 0.012); no significant effects were observed for sleep, depression, or inflammatory markers.	Sig; High
Chadda et al., 2024 [39]	UK	Cross- sectional	N = 392 Age 42 (30–50) y 73% female	From the COVID-19 convalescent immunity study	Biomarker classification: vitamin D deficiency defined as <30 nmol/L	Symptom presence, onset, duration	Self-report (8 symptoms including fatigue, breathlessness, etc.)	Vitamin D deficiency (15.6% prevalence) was associated with a greater number of symptoms (*p* = 0.003), prolonged body aches (OR = 3.07; *p* = 0.001), fatigue (OR = 2.09; *p* = 0.027).	Sig; Moderate
Kaddah et al., 2024 [40]	Egypt	Cross- sectional	N = 84 Age 40.5 ± 14.0 y 72.6% female	3–6 months post-COVID-19 infection	Biomarker assessment: serum 25(OH)D levels	Post-COVID-19 symptoms	CFS, mMRC dyspnea scale, CRP	Vitamin D insufficiency (44%) and deficiency (36%) were not significantly associated with post-COVID-19 symptoms or outcomes.	Non-sig; Low
Wu et al., 2024 [41]	Taiwan	Retrospective	N = 16,600 Age 49.5 ± 17.6 y 66.4% female	ICD-10 code U09 TriNetX research network	Biomarker: vitamin D deficiency (<20 ng/mL) vs. non-deficient (≥20 ng/mL)	ED visits, hospitalization, mortality, post-COVID-19 diagnosis	TriNetX database; ICD-10 U09	VDD was not associated with post-COVID-19 diagnosis (HR = 0.980; 95% CI: 0.630–1.523).	Non-sig; High
Atieh et al., 2025 [42]	USA	RCT	N = 151. Age 45 ± 12.9 y 70.4% female	≥2 moderate symptoms >3 months	Supplement intervention: Vitamin K2 (240 µg/day) + vitamin D3 (2000 IU/day) vs. standard care for 24 weeks	Long COVID-19 symptom burden and diagnosis	RECOVER long COVID-19 Research Index (threshold ≥12); number and type of symptoms	Vitamin K2/D3 reduced RECOVER Index by 7.1% vs. a 7.2% increase in controls (*p* = 0.01); symptoms remained stable in the intervention group but worsened in controls (*p* = 0.03).	Sig; High
Chung et al., 2023 [43]	China (Hong Kong)	RCT Open-label	N = 24 Age 36 (26.0–43.0) y 56% female	olfactory dysfunction (OD) post-COVID-19.	Supplement intervention: Vitamin A (25,000 IU/day) with or without aromatherapy OT vs. clinical observation for 4 weeks	Olfactory function (primary), smell identification, olfactory bulb/tract volume, and olfactory cortical network connectivity	Butanol Threshold Test (≥2-point increase = clinical improvement); imaging and functional connectivity analysis	Oral vitamin A combined with olfactory training significantly improved olfactory function vs. training alone and control (BTT score, *p* < 0.001); also increased olfactory network activity.	Sig; Moderate
Tehrani et al., 2024 [44]	Iran	RCT Open-label	N = 66 Age 49.35 ± 13.83 y 47% female	Persistent symptoms ≥3 weeks after onset	Supplement intervention: Vitamin B1 (600 mg/day) + supportive therapy vs. supportive therapy alone for 8 weeks	Symptom severity (e.g., fatigue, anosmia, sleep disorders); sleep quality	0–10 Visual Assessment Tool (weekly); PSQI	Vitamin B1 led to greater symptom resolution from week 6 onward; by week 8, 87% recovered vs. 40% in controls (*p* < 0.0001).	Sig; Moderate

Notes: 25(OH)D (25-hydroxyvitamin D), 6MWT (Six-Minute Walk Test), BDI (Beck Depression Inventory), BTT (Butanol Threshold Test), CFS (Chalder Fatigue Scale), CI (Confidence Interval), CRP (C-reactive protein), HR (Hazard Ratio), ICD-10 (International Classification of Diseases, 10th Revision), IL-6 (Interleukin-6), IU (International Units), mg (milligrams), MMD (Major Mood Disorder), NICE (National Institute for Health and Care Excellence), OR (Odds Ratio), OT (Olfactory Training), PCQ (Post-COVID-19 Questionnaire), PSQI (Pittsburgh Sleep Quality Index), QoL (Quality of Life), RCT (randomized controlled trial), RECOVER Index (Long COVID-19 symptom burden measure), SF-36, 36-Item Short Form Health Survey, VDD (Vitamin D Deficiency).

**Table 3:** Summary of studies on multinutrient, nutraceutical, and combined formulations for long COVID-19 management and recovery.

Study, Year, Ref.	Country	Study Design	Study Sample	Long COVID-19 Assessment	Type of Nutritional Exposure	Outcomes Assessed	Assessment Tools/ Criteria	Main Results	Significance and Strength of Evidence
Rossato et al., 2021 [45]	Italy	Pre-post Intervention	N = 201 Age 48.11 ± 13.16 y 60.7%, female	Persistent fatigue following infection, with a median of 37 days since disease onset	Supplement intervention: Apportal^®^ (daily sachet containing 19 nutrients including B vitamins, minerals, amino acids, and plant extracts) for 28 days	QoL, fatigue, mental fatigue	EQ-5D index, VAS, FACIT-Fatigue, modified Chalder Q (baseline, day 14 and 28)	Significant improvements in QoL and fatigue at days 14 and 28 (*p* < 0.0001); 76.6% improved FACIT-F by day 14	Sig; Moderate
Naureen et al., 2021 [46]	Italy	Pilot Study	N = 40 Age 52.25 ± 12.0 y	Self-reported fatigue persisting after recovery from COVID-19	Combination supplement: Acetyl L-carnitine, hydroxytyrosol, vitamins B1, B6, B9, B12, C, and D3	Perceived fatigue, tension, energy, calmness	AD-ACL; 15-day pre-post	123% ↑ in energy, ~50% ↓ in tiredness/tension in supplement users; no significant change in controls	Sig; Low
Landi et al., 2022 [47]	Italy	Intervention vs. Control	N = 66 Age 61.0 ± 11.8 y 44%, female	Experiencing fatigue, with a mean of 94.2 ± 22.3 days since onset	Supplement intervention: Amino-Ther Pro (10 amino acids + vitamins B1 and B6 + organic acids), 2 servings/day for 8 weeks vs. no supplement	Muscle index, handgrip, chair-stand, 6MWT, QoL	Handgrip dynamometry, 1-minute chair-stand, 6MWT, EuroQol, VAS	Significant improvements in physical function and muscle metrics; gains remained significant after adjustment	Sig; Moderate
Galluzzo et al., 2022 [48]	Italy	Pre-post Intervention	N = 30 Age 56.14 ± 13.9 y 70%, female	Fatigue and reduced exercise tolerance 30–90 days after COVID-19 diagnosis.	Supplement intervention: Apportal^®^ (same formulation as above), daily for 28 days	Muscle strength, performance, body composition, inflammation	Handgrip, sit-to-stand test, BIA (phase angle, SMI, fat mass), CRP, VAS	Increased handgrip (26.3→28.9 kg), strength time, chair-stands (22→28.3), phase angle, and VAS (all *p* < 0.05)	Sig; Low
Kharaeva et al., 2022 [49]	Slovenia	RCT	N = 160 Age 38–69 y 60.6%, female	Post-COVID-19 following moderate or severe infection	Supplement intervention: Fermented Carica papaya and *Morinda citrifolia* extract vs. placebo for 20 days	Post-COVID-19 symptoms, inflammation	Self-reported symptoms, IL-6, TNF-α assays	Reduced fatigue, joint pain, cognitive symptoms; decreased IL-6, TNF-α	Sig; High
Gaylis et al., 2022 [50]	USA/ Israel	RCT Open-label	N = 51 Age 21–73 y 66.7%, female	symptoms ≥3 months	Supplement intervention: Nutraceutical blend (β-caryophyllene, pregnenolone, DHEA, quercetin, bromelain, zinc, vitamin D), twice daily for 4 weeks	Symptom severity, global well-being	Self-reported symptom checklist (12 items), global rating scale	72–84% reported symptom improvement; 88% showed overall benefit	Sig; Low
Tomasa- Irriguible et al., 2024 [51]	Spain	Double-blind RCT	N = 246 Age 46.8 ± 16.3 y 68%, female	Outpatients with acute COVID-19	Supplement intervention: MMS tablet (13 nutrients including vitamins A, B6, B12, C, D3, E, folic acid, zinc, selenium, iron, copper) vs. placebo for 14 days	Long COVID-19 incidence, cognition, QoL	ICD diagnosis tracking (6 months), MoCA-BLIND, EQ-5D-5L	No effect on long COVID-19 incidence; non-significant cognitive benefit; no QoL differences	Non-sig; High
Noce et al., 2024 [52]	Italy	Double-blind RCT	N = 33 Age 47.6 ± 16.y 57.6%, female	Long COVID-19 patients time since infection 73.7 ± 35.9 days	Supplement intervention: OFS (Echinacea angustifolia, rosehip, propolis, royal jelly, zinc, vitamin C, polyphenols) vs. placebo for 2 months	Inflammation, vitamin D, fatigue, QoL	CRP (lab), serum vitamin D, Fatigue Severity Scale, QoL scale	Reduced CRP (*p* = 0.0145), increased vitamin D (*p* = 0.0005), improved fatigue and QoL	Sig; Moderate
Marra et al., 2024 [53]	Italy	Retrospective	N = 44 Age 49.1 ± 18.1 y 56.8%, female	at least one persistent symptom consistent with post-COVID-19 syndrome	Supplement intervention: PIRV-F20^®^ (lactoferrin, lysozyme, Lactobacillus, resveratrol, vitamins A, C, D3, E, K2, zinc, copper) vs. control for 6 weeks	6MWT, handgrip, symptoms, cardiac function	6MWT, handgrip dynamometry, symptom report, cardiac evaluation (not specified)	6MWT improved more in intervention group (+40 m vs. +10 m, *p* = 0.01); no difference in strength or cardiac function	Sig; Low

Notes: 6MWT (Six-Minute Walk Test), AD-ACL, Activation-Deactivation Adjective Check List, BIA (Bioelectrical Impedance Analysis), CRP (C-reactive protein), DHEA (Dehydroepiandrosterone), EQ-5D (EuroQol 5-Dimension quality of life scale), FACIT-F (Functional Assessment of Chronic Illness Therapy–Fatigue), ICD (International Classification of Diseases), IL-6 (Interleukin-6), MMS (Micronutrient and Multivitamin Supplement), MoCA-BLIND (Montreal Cognitive Assessment—vision-independent version), OFS (Oral Formulation Supplement: Echinacea, rosehip, propolis, etc.), QoL (Quality of Life), RCT (randomized controlled trial), SMI (Skeletal Muscle Index), TNF-α (Tumor Necrosis Factor alpha), VAS (Visual Analog Scale).

**Table 4:** Summary of studies investigating amino acids and metabolic support interventions for long COVID-19 fatigue and recovery.

Study, Year, Ref.	Country	Study Design	Study Sample	Long COVID-19 Assessment	Type of Nutritional Exposure	Main Study Outcome	Assessment Tools/Criteria	Main Results	Significance and Strength of Evidence
Tosato et al., 2022 [54]	Italy	Single-blind RCT	N = 46 Age 50.5 ± 14.0 y 65.2% female.	WHO criteria + persistent fatigue responding “most or all the time” to item seven of the CES-D (“I felt that everything I did was an effort”)	Supplement intervention: L-arginine (1.66 g) + liposomal vitamin C (500 mg), twice daily vs. placebo for 28 days	Physical function, endothelial function, fatigue	6MWT, handgrip dynamometry, flow-mediated dilation, CES-D fatigue item	Intervention group showed greater gains in walking (+30 m), handgrip (+3.4 kg), and dilation (14.3% vs. 9.4%); fatigue reported by 8.7% vs. 80.1% in placebo (all *p* < 0.05)	Sig; Moderate
Cash et al., 2022 [55]	USA	Non-randomized CT	N = 43 Age 47 years; 73.7% female.	long COVID-19 experienced at least 6 months of fatigue with no prior fatigue	Supplement intervention: Anhydrous Enol-Oxaloacetate (500 or 1000 mg, twice daily) for 6 weeks	Fatigue	Chalder Fatigue Questionnaire, PROMIS Fatigue 7A, Fatigue Severity Scale	Fatigue scores decreased by up to 46.8% at 6 weeks	Sig; Low
Izzo et al., 2022 [56]	Italy	Observational (LINCOLN Survey)	N = 1390 Age 55.5 ± 15.7 y 49.5% female	Presence of COVID-19 sequelae that extend beyond four weeks after initial infection.	Supplement comparison: L-arginine (1.66 g) + liposomal vitamin C (500 mg) vs. multivitamins (B1, B2, B6, B12, folic acid, niacin, pantothenic acid) for 30 days	Long COVID-19 symptom burden, physical exertion	Symptom checklist; modified Borg scale (0–10)	L-arginine + vitamin C group had lower symptom scores (8.15 ± 1.3 vs. 13.9 ± 2.3; *p* < 0.001) and Borg effort scores (*p* < 0.0001)	Sig; Moderate
Turcu-Stiolica et al., 2023 [57]	Romania	Prospective	N = 505 Age 50(39–63) y 54.3% female	SARS-CoV-2 infection with mental and/or physical fatigue during or after illness.	Supplement intervention: Astenor Energy^®^ or Astenor Forte^®^ based on liver enzyme status, 10 days/month for 3 months	Fatigue	FAS	FAS scores improved in both groups (median 33→17 and 25→17; *p* < 0.0001); fatigue type varied by group	Sig; Moderate
Slankamenac et al., 2023 [58]	Serbia	Double-blind RCT	N = 12 Age 27.5 ± 6.8 y 50% female	COVID-19~3 months, fatigue 20-MFI score >43.5) +1 symptom	Supplement intervention: creatine monohydrate (4 g/day) vs. placebo for 6 months	Muscle creatine, fatigue, symptoms, endurance	1.5T MRS, MFI-20, symptom VAS, treadmill time-to-exhaustion	Creatine increased brain/muscle creatine (*p* < 0.05), reduced fatigue (*p* = 0.04), and improved time to exhaustion (+65 s); large symptom effect sizes (d = 1.26–3.03)	Sig; Low
Slankamenac et al., 2024 [59]	Serbia	Double-blind RCT	N = 15 Age 39.7 ± 16.0 y 60%, females	long COVID-19 with fatigue and at least one other long COVID-19 symptom	Supplement comparison: creatine (8 g/day) ± glucose (3 g/day) vs. glucose alone, for 8 weeks	Brain/muscle creatine, fatigue, symptoms, endurance	MRS, MFIS-20, symptom VAS, treadmill test	Creatine (±glucose) increased brain creatine (*p* < 0.05), improved exhaustion time (+205 s; *p* = 0.03), and reduced fatigue (*p* = 0.008)	Sig; Low

Note: 6MWT (Six-Minute Walk Test), CES-D (Center for Epidemiologic Studies Depression Scale), CT (Controlled Trial), FAS (Fatigue Assessment Scale), MFI-20 (Multidimensional Fatigue Inventory–20), MFIS-20 (Modified Fatigue Impact Scale–20), MRS (Magnetic Resonance Spectroscopy), PROMIS Fatigue 7A (Patient-Reported Outcomes Measurement Information System Fatigue Short Form 7A), RCT (randomized controlled trial), VAS (Visual Analog Scale), WHO (World Health Organization).

**Table 5:** Summary of studies investigating gut microbiota modulation for symptom management in long COVID-19.

Study, Year, Ref.	Country	Study Design	Study Sample	Long COVID-19 Assessment	Type of Nutritional Exposure	Main Study Outcome	Assessment Tools/Criteria	Main Results	Significance and Strength of Evidence
Thomas et al., 2022 [60]	UK	Double-blind RCT	N = 147 Age 53 y 44%, female	Long COVID-19 mean duration of symptoms: 108 days	Supplement intervention: phytochemical-rich capsule (curcumin, chamomile, citrus bioflavonoids, pomegranate, resveratrol) + probiotic (Lactobacillus + inulin) vs. placebo for 28 days	Fatigue, cough, well-being	CFS, Cough Symptom Score, Subjective Well-being Score	Intervention group had 2× greater fatigue reduction, 3× cough improvement, and >2× well-being gains vs. placebo (*p* = 0.02)	Sig; High
Docampo et al., 2024 [61]	Switzerland	Pre-post Intervention	N = 6 Age 31.7 ± 16 y 67% female	Long COVID-19 over 12 months with symptoms of fatigue, cognitive difficulties, and dizziness.	Supplement intervention: Paraprobiotic formula (Abiprol + Brexibiol) twice daily for 4 weeks	Fatigue, QoL, dysautonomia, depression, digital activity, immune markers	CFS, Bell Disability Scale, SF-36, COMPASS 31, PHQ-9, smartphone/wearable data, immune profiling	30–80% symptom improvement across domains; reduced TLR2/CD40/HLA-DR immune activation; better sleep and fatigue in most participants	Sig; Low
Lau et al., 2024 [62]	China (Hong Kong)	Double-blind RCT	N = 463 Age 49·3 ±13 y 66%, female	CDC criteria+ at least one symptom from the PACSQ-14 persisting for ≥4 weeks after infection.	Supplement intervention: synbiotic (SIM01 with Bifidobacteria + prebiotics) vs. placebo	PACS symptoms (fatigue, cognitive, GI, general unwellness)	Symptom checklist; odds ratios for symptom alleviation	Significantly greater alleviation of fatigue (OR = 2.27), memory loss, concentration difficulty, GI symptoms, and general unwellness (all *p* < 0.01)	Sig; High
Calvani et al., 2024 [63]	Italy	Double-blind RCT	N = 31 Age 50.3 ± 12.9 y 45.5%, female	WHO criteria, with persistent fatigue defined as responding “most or all the time” to item 7 of the CES-D scale (“I felt that everything I did was an effort”).	Supplement intervention: beetroot juice (600 mg nitrate/day) vs. placebo for 14 days	Fatigue, physical function, vascular response, microbiota, inflammation	Fatigue resistance test, 6MWT, handgrip, FMD, gut microbiota, cytokine panels	Both groups improved from baseline in fatigue and 6MWT; beetroot group had gut microbiota shifts and increased IFN-γ, MIP-1β (no group differences in primary outcomes)	Non-sig; Moderate
Cuevas- Sierra et al., 2024 [64]	Spain	Cross- Sectional	N = 188 Age 49 ± 0.9 y 87%, female	Diagnosed with long COVID-19 by the internist doctor	Dietary pattern: high vs. low Mediterranean diet adherence (cutoff ≥7 points)	Inflammation, oxidative stress, gut microbiota	Blood biomarkers (LDL, glucose, LDH), redox indices, microbiota analysis	High MD adherence linked to better metabolic profile, redox balance, and lower Oscillibacter abundance; stronger effects with high olive oil intake	Sig; Moderate
Ranisavljev et al., 2025 [65]	Serbia	Double-blind RCT	N = 26 Age 42.5 ± 13.4 y 50%, female	COVID-19~3 months, fatigue 20-MFI score >43.5) +1 symptom	Supplement intervention: daily synbiotic (*L. rhamnosus*, *L. plantarum*, *B. lactis*, *B. longum*, FOS, zinc) for 3 months	Fatigue, post-exertional malaise, brain/muscle metabolism, endurance	20-MFI, VAS, treadmill test, brain MRS (tCho, tCr, NAA)	Improved post-exertional malaise, increased brain choline/creatine, reduced fatigue, and extended time to exhaustion	Sig; Moderate

Notes: 20-MFI (20-item Multidimensional Fatigue Inventory), CES-D (Center for Epidemiologic Studies Depression Scale), CDC (Centers for Disease Control and Prevention), CFS (Chalder Fatigue Scale), COMPASS 31 (Composite Autonomic Symptom Score—31 items), FMD (Flow-Mediated Dilation), FOS (Fructooligosaccharides), GI (gastrointestinal), IFN-γ (Interferon gamma), LDH (Lactate Dehydrogenase), LDL (Low-Density Lipoprotein), MD (Mediterranean Diet), MFI (Multidimensional Fatigue Inventory), MIP-1β (Macrophage Inflammatory Protein 1 beta), MRS (Magnetic Resonance Spectroscopy), NAA (N-acetylaspartate), OR (Odds Ratio), PACSQ-14 (Post-Acute COVID-19 Symptom Questionnaire—14 items), PHQ-9 (Patient Health Questionnaire–9), QoL (Quality of Life), RCT (randomized controlled trial), SF-36 (Short Form Health Survey–36), SIM01 (Synbiotic formulation name), VAS (Visual Analog Scale), WHO (World Health Organization).

**Table 6:** Summary of studies on lifestyle, nutritional status, and interventions related to risk and recovery in long COVID-19.

Study, Year, Ref.	Country	Study Design	Study Sample	Long COVID-19 Assessment	Type of Nutritional Exposure	Main Study Outcome	Assessment Tools/Criteria	Main Results	Significance and Strength of Evidence
Pre-Infection Lifestyle Factors and Risk of long COVID-19									
Wang et al., 2023 [66]	USA	Prospective cohort (Nurses’ Health Study II)	N = 32,249 Age 64.7 ± 4.6 y 100%, female	1981 tested positive for SARS-CoV-2	Healthy lifestyle factors pre-COVID-19 (0–6 score)	Post-COVID-19 condition (PCC) risk	Self-reported PCC (≥4 weeks)	5–6 healthy factors linked to 49% lower PCC risk (RR = 0.51); BMI and sleep showed independent associations	Sig; High
Wang et al., 2024 [67]	UK	Prospective cohort study (UK Biobank)	N = 68,896 Age 68.1 ± 8.1 y 44.7%, female	Confirmed SARS-CoV-2 infection from the UK Biobank cohort	Lifestyle factors pre-infection (diet, BMI, PA, etc.)	Post-COVID-19 sequelae, death, hospitalization	Health records and lifestyle surveys	Favorable lifestyle (6–10 factors) linked to lower risk of sequelae (HR = 0.64), mortality (HR = 0.59), and hospitalization (HR = 0.78)	Sig; High
Prolong Nutritional status after COVID-19									
Gérard et al., 2021 [68]	France	Prospective cohort	N = 288 Age 59.8 ± 16.6 y 45.8%, female	Post-hospitalized COVID-19 patients at 6 months with sequelae	Nutrition support (ONS, diet, activity) in post-hospital patients	Malnutrition, performance, fatigue	GLIM, SES, VAS	36% still malnourished at 6 months; ICU stay and obesity predicted worse outcomes	Sig; Moderate
Álvarez-Hernández et al., 2023 [69]	Spain	Prospective cohort	N = 199 Age 60.7 ± 10.1 y 29.6%, female	COVID-19 ICU survivors evaluated 3, 6, and 12 months after discharge.	Nutrition support (ONS, energy/protein) post-ICU	Malnutrition risk, physical function, sarcopenia	MUST, EQ-5D-3L, SARC-F	35% at risk after 1 year; 25% had sarcopenia risk; low intake common; support linked to better outcomes	Sig; Moderate
Lakenman et al., 2023 [70]	Netherlands	Prospective	N = 48 Age 60 [52; 65] y 73% male	COVID-19 ICU survivors evaluated 1-year post-discharge.	Protein/energy intake at 1-year post-ICU	Nutrition status, strength, body comp.	MUST, FFMI, FMI, GLIM, handgrip	Weight regained but 50% had high fat mass; protein intake suboptimal despite no malnutrition	Non-sig (mixed); Low–Moderate
Muzaffar et al., 2024 [71]	Malaysia	Case– Control	N = 108 Age 21.06 ± 1.37 y 74.1%, female	*n* = 54 COVID-19-recovered vs. *n* = 54 healthy controls	Diet quality in COVID-19-recovered vs. controls	Diet diversity, sleep, weight	DQQ Malaysia, sleep self-report	COVID-19 group had lower diet diversity; no significant sleep–weight associations	Non-sig; Low
Mejía Alonso et al., 2024 [72]	Mexico	Correlational follow-up	N = 66 Age 51.3 y 25% female	Hospitalized for COVID-19 and referred to rehabilitation >139 days post-discharge.	Nutrition protocol and counseling in rehab patients	Nutritional recovery, muscle mass/function	Ultrasound (muscle), HRQoL, mobility tests	Improved muscle strength, mass, respiratory function; excess weight reduced 6MWT performance	Sig; Low–Moderate
Multi-professional intervention and diet modification in Post-COVID-19 Recovery									
Sousa-Catita et al., 2022 [73]	Portugal	Prospective intervention study	N = 118 Age 71.9 (41–90) y 52%, female	Post-COVID-19 patients admitted to rehabilitation care units > 30 days.	Interdisciplinary rehab (ESPEN-based nutrition, exercise, therapy)	Nutrition and functional status	BMI, MUAC, MNA^®^, HGS	BMI, HGS, and MNA^®^ improved; largest gains in ICU group and final 15 days of rehab	Sig; Moderate
Nikolic Turnic et al., 2022 [74]	Serbia	Cross- Sectional	N = 80 Age 30.6± 1.5 y 75%, female	Working-age adults (mostly under 30, with confirmed COVID-19 prior 6 months	Self-reported diet and lifestyle 6 months post-COVID-19	QoL, dietary change, activity	pCOVq, WHOQOL-BREF	Water intake ↑; no major nutrient changes; reduced physical/social activity; slight QoL decline	Non-sig (lifestyle only); Low
Sordi et al., 2023 [75]	Brazil	Non- randomized CT	N = 35 Age 42 ± 12 y 90% male	Overweight adults post-COVID-19 (BMI ≥25), self-reported post-COVID-19 symptoms,	8-week group program: nutrition + exercise + education	Body comp, fitness, biomarkers	CRP, glucose, HDL-c, strength tests	Strength, flexibility, inflammation, and metabolic markers improved, esp. in moderate/severe cases	Sig; Low–Moderate
Ryal et al., 2023 [76]	Brazil	Controlled trial with parallel groups and repeated measures	N = 55 Age 49 ± 13 y 65.4% male	Middle-aged overweight or obese COVID-19 survivors with self-reported post-COVID-19 symptoms	8-week intervention: nutrition + mental health + exercise	Mental health (anxiety, depression, PTSD, well-being)	GAD-7, PHQ-9, IES-R, MHC-SF	Mental health improved most in mild/control groups; less consistent gains in moderate/severe groups	Sig; Moderate
Pink et al., 2024 [77]	Germany	RCT (Secondary analysis)	N = 92 Age 47 (40, 53) y 75% female	Post-COVID-19 patients with fatigue FAS ≥22 matched to healthy controls ratio 1:1	Diet behavior (7-day diary), omega-3/6, sleep	Fatigue, mental health, nutrient intake	FAS, SF-36, sleep/activity tracking	PASC group had healthier diet trends, longer sleep (+49 min); no intake/activity differences	Non-sig; Moderate
Combet et al., 2025 [78]	UK	RCT Open-label	N = 234 Age 46.4 ± 9.1 y 84.5%, female	long COVID-19 with symptoms >12 weeks) and BMI >27 kg/m^2^	12-week remote weight-loss program (diet replacement + reintro)	Long COVID-19 symptom change	VAS, symptom questionnaires	Intervention led to greater symptom improvement (mean diff = –0.34; 95% CI: –0.67 to –0.01)	Sig; High
Suárez-Moreno et al., 2025 [19]	Spain	Cross- Sectional	N = 305 Age 52.8 ± 11.9 y 68%, female	WHO’s definition of long COVID-19. History of different SARS-CoV-2 infection symptoms >3 months after onset.	Mediterranean diet adherence (assessed via MEDAS)	Metabolic syndrome components	BMI, HDL-c, waist, uric acid link to long COVID-19	Higher MD adherence linked to lower BMI, waist, uric acid, and MetS risk; higher HDL	Sig; Moderate

**Notes:** BMI (Body Mass Index), CI (Confidence Interval), CRP (C-reactive Protein), DQQ (Dietary Quality Questionnaire), EQ-5D-3L (EuroQol 5-Dimension, 3-Level), ESPEN (European Society for Clinical Nutrition and Metabolism), FAS (Fatigue Assessment Scale), FFMI (Fat-Free Mass Index), FMI (Fat Mass Index), GAD-7 (Generalized Anxiety Disorder 7-item scale), GLIM (Global Leadership Initiative on Malnutrition), HGS (Handgrip Strength), HDL-c (High-Density Lipoprotein Cholesterol), HR (Hazard Ratio), HRQoL (Health-Related Quality of Life), IES-R (Impact of Event Scale–Revised), MEDAS (Mediterranean Diet Adherence Screener), MetS (Metabolic Syndrome), MHC-SF (Mental Health Continuum—Short Form), MNA^®^ (Mini Nutritional Assessment), MUAC (Mid-Upper Arm Circumference), MUST (Malnutrition Universal Screening Tool), ONS (Oral Nutritional Supplementation), PA (Physical Activity), pCOVq (Post-COVID-19 Questionnaire), PHQ-9 (Patient Health Questionnaire–9), QoL (Quality of Life), RCT (randomized controlled trial), RR (Relative Risk), SARC-F (Sarcopenia Screening Questionnaire), SF-36 (Short Form Health Survey–36), VAS (Visual Analog Scale), WHO (World Health Organization), WHOQOL-BREF (World Health Organization Quality of Life—BREF version.

**Table 7:** Summary of key nutritional exposures and supporting evidence in long COVID-19 recovery and management.

Type of Nutritional Exposure	No. of Studies	% Reporting Significant Findings	General Strength of Evidence	Details in Table
Vitamin D (e.g., Biomarker, deficiency, supplement)	10	40%	Mostly moderate	Table 2
Vitamin D with Zinc/Magnesium/ Vitamin K_2_	3	100%	Low-to-high	
Vitamins A/ B1	2	100%	Moderate	
Multinutrient/Nutraceutical (e.g., Apportal^®^, MMS, OFS, fermented papaya extract, and blends with vitamins, amino acids, minerals, and polyphenols)	9	89%	Low-to-Moderate	Table 3
Amino Acids/Metabolic Support (e.g., L-arginine + vitamin C, oxaloacetate, creatine, and Astenor formulations with amino acids and cofactors)	6	100%	Low-to-Moderate	Table 4
Gut Microbiota-Targeted Therapies (e.g., SIM01 synbiotic, paraprobiotics, beetroot juice, phytochemical + probiotic blends, and Mediterranean diet adherence)	6	83%	Moderate-to-high	Table 5
Pre-Infection Lifestyle Factors (e.g., healthy diet, BMI and other modifiable risk factors)	2	100%	High	Table 6
Nutritional Status / Deficiency Studies (e.g., oral nutrition support, protein/energy intake, diet quality, and post-ICU malnutrition risk)	5	60%	Low-to-Moderate	
Multicomponent Lifestyle Interventions (e.g., diet combined with physical activity, mental health support, weight loss, or rehabilitation programs)	7	71%	Mostly moderate	

Note: BMI (Body Mass Index), ICU (Intensive Care Unit), MMS (Multiple Micronutrient Supplement), OFS (Oligofructose Supplement), SIM01 (Synbiotic Intervention Mix 01).

## Abbreviations

The following abbreviations are used in this manuscript:

20-MFI	20-item Multidimensional Fatigue Inventory
25OHD	25-hydroxyvitamin D
6MWT	6-Minute Walk Test
7-day food diary	Seven-day dietary intake recording tool
ADMA	Asymmetric Dimethylarginine
AEO	Enol-Oxaloacetate
BDI	Beck Depression Inventory
BioICOPER	Biopsychosocial Integrated COVID Pathway for Evaluation and Rehabilitation
BMI	Body Mass Index
BTT	Butanol Threshold Test
CDC	Centers for Disease Control and Prevention
CES-D	Center for Epidemiologic Studies Depression Scale
CFS	Chalder Fatigue Score
COMPASS 31	Composite Autonomic Symptom Score 31
COVID-19	Coronavirus Disease 2019
CRP	C-reactive protein
CT	Controlled Trial
DQQ	Dietary Diversity Score
DSM	Diagnostic and Statistical Manual of Mental Disorders
ED	Emergency Department
EQ-5D	EuroQol 5-Dimension Questionnaire
EQ-5D-5L	EuroQol 5-Dimension 5-Level Questionnaire
ESPEN	European Society for Clinical Nutrition and Metabolism
FACIT	Functional Assessment of Chronic Illness Therapy
FAS	Fatigue Assessment Scale
FFMI	Fat-Free Mass Index
FMI	Fat Mass Index
GAD-7	Generalized Anxiety Disorder—7 item scale
GLIM	Global Leadership Initiative on Malnutrition
HR	Hazard Ratio or Heart Rate (context-dependent)
HRQoL	Health-Related Quality of Life
ICD	International Classification of Diseases
ICU	Intensive Care Unit
IES-R	Impact of Event Scale—Revised
IL-6	Interleukin-6
IQR	Interquartile Range
IU	International Unit
LDH	Lactate Dehydrogenase
LDL	Low-Density Lipoprotein
MD	Mediterranean Diet
MDPI	Multidisciplinary Digital Publishing Institute
MeSH	Medical Subject Headings
MEDAS	Mediterranean Diet Adherence Screener
ME/CFS	Myalgic Encephalomyelitis/Chronic Fatigue Syndrome
MetS	Metabolic Syndrome
MHC-SF	Mental Health Continuum—Short Form
MLR	Monocyte-to-lymphocyte ratio
MMS	Multiple Micronutrient Supplement
MoCA-BLIND	Montreal Cognitive Assessment—Blind Version
MNA	Mini Nutritional Assessment
NHS	National Health Service
NICE	National Institute for Health and Care Excellence
NLR	Neutrophil-to-lymphocyte ratio
NNLM	National Network of Libraries of Medicine
OFS	Oral Food Supplement
Omega-3/6	Omega-3 and Omega-6 polyunsaturated fatty acids
ONS	Oral Nutritional Supplements
OR	Odds Ratio
PACS	Post-Acute COVID-19 Syndrome
PCC	Post-COVID-19 Condition
pCOVq	Post-COVID-19 Questionnaire
PCQ	Primary Care Questionnaire (or Patient Care Quality; context-dependent)
PHQ-9	Patient Health Questionnaire-9
PACSQ-14	Post-Acute COVID-19 Syndrome Questionnaire
PREDIMED	Prevención con Dieta Mediterránea / Prevention with Mediterranean Diet
PRISMA	Preferred Reporting Items for Systematic Reviews and Meta-Analyses
PRISMA-ScR	Preferred Reporting Items for Systematic Reviews and Meta-Analyses Extension for Scoping Reviews
PROMIS	Patient-Reported Outcomes Measurement Information System
PSQI	Pittsburgh Sleep Quality Index
QoL	Quality of life
RCT	randomized controlled trial
SARC-F	Strength, Assistance with walking, Rise from a chair, Climb stairs, and Falls
SARS-CoV-2	Severe Acute Respiratory Syndrome Coronavirus 2
SF-36	36-Item Short Form Survey
SIM01	A specific probiotic strain/formulation (context-dependent)
TDR	Total Diet Replacement
UK	United Kingdom
VAS	Visual Analogue Scale
VDD	Vitamin D deficiency
WHO	World Health Organization
WHOQOL-BREF	World Health Organization Quality of Life—Brief Version
